# Combined Exposure to Fructose and Bisphenol A Exacerbates Abnormal Lipid Metabolism in Liver of Developmental Male Rats

**DOI:** 10.3390/ijerph16214152

**Published:** 2019-10-28

**Authors:** Ren Lin, Yue Jia, Fengjuan Wu, Yuan Meng, Qi Sun, Lihong Jia

**Affiliations:** Department of Child and Adolescent Health, School of Public Health, China Medical University, Shenyang 110122, China; 15804066682@163.com (R.L.); dianalisaj@sina.com (Y.J.); 18524430655@163.com (F.W.); yuanyuan_max@163.com (Y.M.); sunqi@cmu.edu.cn (Q.S.)

**Keywords:** fructose, bisphenol A, lipid metabolism, inflammatory response

## Abstract

The aim of this study was to investigate whether combined exposure to fructose and bisphenol A (BPA) has a synergistic effect on abnormal lipid metabolism in the liver of developmental male rats and its possible mechanism. Fifty weaned male Wistar rats were divided into five groups: the control, 13% fructose, 20% fructose, 1 µg/mL BPA, and 13% fructose + 1 µg/mL BPA (combined exposure). Rats were exposed to fructose and/or BPA through drinking water for eight weeks. Genes or proteins regulating lipid metabolism include sterol regulatory element binding protein 1 (SREBP1), adipose triglyceride lipase (ATGL), hormone sensitive lipase (HSL), acetyl-CoA carboxylase 1 (ACC1), fatty acid synthase (FAS), zinc α 2 glycoprotein (ZAG) and estrogen receptor α (ERα), and the expression of proteins regulating inflammatory response, such as TLR4 and NF-κB, were determined. Serum total cholesterol (T-CHO), triglyceride (TG), low, high density lipoprotein cholesterol (LDL-C, HDL-C), blood glucose, insulin, IL-17 and TNF-α levels were also measured. Liver tissue morphology was observed by H&E staining. The results showed that the levels of gene and protein catalyzing lipogenesis were increased (SREBP1, ACC1 and FAS), while those catalyzing lipolysis were decreased (ATGL, HSL and ZAG), accompanied by dyslipidemia, insulin resistance and hepatic fat accumulation, and there were higher expression of TLR4 and NF-κB protein and lower expression of ERα protein in liver, and increased serum IL-17 and TNF-α levels in fructose and/or BPA exposed rats compared with controls. Moreover, the above indicators were more serious in combined exposure group than in single exposure group. Therefore, abnormal lipid metabolism in the liver of developmental rats could be exacerbated by combined exposed to fructose and BPA.

## 1. Introduction

In recent years, the prevalence of obesity and dyslipidemia has increased rapidly, which causes decreased quality of life and increased mortality due to their roles as important risk factors of cardiovascular and cerebrovascular diseases [1]. Dyslipidemia, characterized by elevated levels of triglyceride (TG) and low-density lipoprotein cholesterol (LDL-C) and decreased levels of high-density lipoprotein cholesterol (HDL-C), was once considered an adult disease, but with the increasing prevalence of childhood obesity, it is becoming more and more common in children and adolescents. It is generally believed that obesity and dyslipidemia are related to heredity, high calorie food intake and less physical activity, etc. 

Epidemiological studies have shown that fructose intake is increasing among people, especially in children and adolescents [2,3]. High fructose consumption, even in the absence of obesity, can cause serious health problems, such as liver fat accumulation, dyslipidemia and nonalcoholic fatty liver disease (NAFLD) [4,5], which may be related to increased hepatic *de novo* lipogenesis (DNL), hepatic endoplasmic reticulum stress and increased expression of lipogenic genes [6,7]. Bisphenol A (BPA), as one of the most produced endocrine disrupting chemicals (EDCs), mainly used in the manufacture of plastics and epoxy resins [8]. BPA can increase fatty acid influx from adipose tissue into the liver and endogenous fatty acid synthesis, which in turn leads to the accumulation of TG in the liver [9]. Increasing evidence has shown that BPA exposure may increase the risk of obesity, insulin resistance and dyslipidemia [10,11]. In addition, the sugar-sweetened beverages (SSBs) preferred by children and adolescents in daily life, contain both fructose and BPA. A report showed that the average concentration of BPA in SSBs was 1.0 ng/mL, while it was 40.3 ng/g in canned foods [12]. In SSBs, fructose constituted 60.6% ± 2.7% of sugar content [13]. It is not clear whether combined exposure to fructose and BPA can aggravate abnormal lipid metabolism, because previous studies have shown that abnormal lipid metabolism can be caused by fructose or BPA.

The liver is the most important organ regulating lipid metabolism. Acetyl-CoA carboxylase 1 (ACC1) and fatty acid synthase (FAS) are the key enzymes to catalyze lipogenesis, while adipose triglyceride lipase (ATGL) and hormone sensitive lipase (HSL) are the key enzymes to catalyze lipolysis. Sterol regulatory element binding protein 1 (SREBP1) is an important transcription factor that regulates many types of key lipogenic genes encoding enzymes, such as ACC1 and FAS, which are involved in TG synthesis and lipid accumulation [14]. Studies have shown that the mechanism of hepatic lipid accumulation induced by fructose or BPA is related to its up-regulation of lipogenic gene expression, such as SREBP1 and FAS [15,16]. However, to our knowledge, the effect of combined exposure to fructose and BPA on the expression of key genes or proteins that regulate lipid metabolism in the liver has not been reported. Hence, we hypothesized that combined exposure to fructose and BPA may exacerbate abnormal lipid metabolism, and its mechanism may be associated with severe interference with the expression of key genes or proteins regulating lipid mechanism. 

Studies have shown that the adverse health effects of excessive fructose or BPA exposure are associated with the activation of inflammatory response [17,18]. Inflammation is the inducement of many chronic diseases, such as diabetes, dyslipidemia, cardiovascular and cerebrovascular diseases [19]. Whether combined exposure to fructose and BPA has a synergistic effect on inflammatory response is unknown. In this study, the nuclear factor κB (NF-κB), as a key inflammatory regulator, toll-like receptor-4 (TLR4), serum interleukin-17 (IL-17) and tumor necrosis factor-α (TNF-α) levels were also measured to verify the more serious adverse effects of combined exposure to fructose and BPA on health.

## 2. Materials and Methods 

### 2.1. Animals

Fifty male Wistar rats aged 3 weeks (80–110 g) were received from the Center for Experimental Animals at China Medical University (Shenyang, China) under National Animal Use License number SCXK-LN 2013-0007. All experiments and surgical procedures were approved by the Animal Use and Care Committee at China Medical University, which complies with the National Institutes of Health Guide for the Care and Use of Laboratory Animals. The rats were housed in a room under controlled temperature (22 ± 2 °C) and relative humidity (55 ± 5%) with a normal 12-h light/dark cycle. Rats were fed standard chow and water *ad libitum* one week for acclimatization before the experiment. After the adaptation period, all rats were kept in a single cage and randomly allocated into five groups according to body weight (*n*= 10/group): control group, 13% fructose group, 20% fructose group, 1 µg/mL BPA group, and 13% fructose + 1 µg/mL BPA group (combined exposure group). Rats were exposed to BPA (99% pure; Sigma-Aldrich, St. Louis, MO, USA) and/or fructose (Shandong Xiwang Sugar, Zibo, China) (400 kcal/100 g) by drinking water alone or in combination and given standard chow (23.7 g protein, 5 g fat and 46.12 g carbohydrate/100 g) for eight weeks.

Food and water intake per cage were measured daily, and body weight was determined once a week. Protein and energy intake were calculated according to the amount of water and/or food consumed. At the end of the study, animals were sacrificed after overnight fasting. Blood was collected from caudal vein, and fasting blood glucose (FBG) levels were measured by using a glucometer (Sinocare, Changsha, China). The blood samples collected from aorta abdominalis were centrifuged (3500 rpm, 4 °C, 5 min) to separate the serum and then stored at −80 °C for use. Liver and visceral fat (epididymal and perirenal fat tissue) were collected and weighed. The visceral fat coefficient or liver coefficient was calculated as visceral fat weight or liver weight/body weight × 100%. Part of the liver was either frozen at −80 °C for next analysis or fixed in 4% paraformaldehyde for further histopathological analysis. 

### 2.2. Serum Analysis

The levels of serum total cholesterol (T-CHO), TG, LDL-C, and HDL-C were measured by using commercial assay kits (Nanjing Jiancheng Bioengineering, Nanjing, China). IL-17 (BioLegend, San Diego, CA, USA), TNF-α, adiponectin (ADP) (Wuhan Boster Biological Technology, Wuhan, China), and insulin (Shanghai Enzyme-linked Biotechnology, Shanghai, China) in serum were measured by ELISA assay kits. All assays were performed according to the manufacturer’s instructions. Homeostasis model assessment of insulin resistance (HOMA-IR) referring to the degree of insulin resistance (IR) was computed according to the following equation: HOMA-IR = FBG (mmol/L) × fasting insulin (mIU/L)/22.5 [20].

### 2.3. Liver Tissue Morphology Observation 

Hepatic pathological changes were observed after hematoxylin and eosin (H&E) staining. Liver tissue were fixed in 10% buffered formalin, dehydrated and embedded in wax. Serial sections of the tissue were taken in an automated microtome at a thickness of 5 µm and stained with H&E reagent. Photomicrographs were captured under a microscope (Leica Microsystems, Werzlar, Germany). The photos were taken a final magnification of 200 ×.

### 2.4. Reverse Transcription Quantitative Real-Time PCR (RT-qPCR)

Total RNA was isolated from liver using Trizol reagent. The cDNA was synthesized from the total RNA by using the PrimeScript RT reagent kit (Takara, Tokyo, Japan). Then the cDNA was served as templates for real-time PCR amplification by using the SYBR^®^ Premix Ex Taq™ II (Takara) and QuantStudio 6 Flex Real-Time PCR System (Life Technologies, Carlsbad, CA, USA). RNA abundance was expressed as 2^−ΔΔCt^ for the target gene normalized against the *GAPDH* gene (as the reference gene), and presented as fold change versus control sample. The following primer pairs were used: *SREBP1*: sense 5’-GTGGTCTTCCAGAGGCTGAG-3’; antisense 5’-GGGTGAGAGCCT TGAGACAG-3’. *ATGL*: sense 5’-AGACTGTCTGAGCAGGTGGA-3’; antisense 5’-AGTAGCTGA CGCTGGCATTC-3’. *HSL*: sense 5’-CTTGTCACATAGGCCCCACT-3’; antisense 5’-GTCACGTGT TGGGTGATCTG-3’. *ACC1*: sense 5’-AGGAAGATGGTGTCCCGCTCTG-3’; antisense 5’-GGGG AGATGTGCTGGGTCAT-3’. *FAS*: sense 5’-CCAAGCAGGCACACACAATG-3’; antisense 5’-GAGT GAGGCCGGGTTGATAC-3’. *GAPDH*: sense 5’-GCAAGAGAGAGGCCCTCAG-3’; antisense 5’-TGTGAGGGAGATGCTCAGTG -3’.

### 2.5. Western Blotting Assay

Preparation of protein sample and western blot analysis were performed according to standard procedures. The concentration of the protein extracted from the livers was quantitated with a BCA protein assay kit (Beijing Dingguo Changsheng Biotechnology, Beijing, China) prior to mixing with loading buffer and heating at 100 °C for 5 min. The extracted protein (50 µg) was separated by SDS-PAGE and then electrophoretically transferred onto polyvinylidene difluoride membranes (Millipore, Bedford, MA, USA). The primary antibodies included: rabbit anti-SREBP1 (NB600-582) purchased from Novus Biological (Littleton, CO, USA) (dilution 1:1000); rabbit anti-FAS (#3180), rabbit anti-NF-κB (#8242), rabbit anti-inhibitor of κB α (IκBα, #4812), rabbit anti-GAPDH (#2118) were procured from Cell Signaling Technology (Danvers, MA, USA) (dilution 1:1000); rabbit anti-HSL (ab45422) purchased from Abcam (Cambridge, MA, USA) (dilution 1:1000); rabbit anti-estrogen receptor α (ERα, sc-8005), rabbit anti-zinc α 2 glycoprotein (ZAG, sc-21720), and rabbit anti-TLR4 (sc-293072) purchased from Santa Cruz Biotechnology (Santa Cruz, CA, USA) (dilution 1:200). Blots were incubated overnight at 4 °C in primary antibody in 5% BSA followed by HRP-conjugated anti-rabbit IgG antibody (abs20002A, Absin Bioscience, Shanghai, China) (dilution 1:5000) or HRP-conjugated anti-mouse IgG antibody (abs20001A, Absin Bioscience) (dilution 1:5000). Specific bands were visualized by ECL detection and quantified via Image J software (Bio-Rad, Hercules, CA, USA).

### 2.6. Statistical Analysis

All analyses were performed with the SPSS 21.0 software (SPSS, Inc., Chicago, IL, USA). Data were expressed as mean ± standard deviations (SD). Differences between groups were analyzed by one-way analysis of variance (ANOVA). For all analyses, *p* < 0.05 was considered statistically significant.

## 3. Results

### 3.1. Effects of Fructose and/or BPA Exposure on Body Weight, Food, Water and Protein Intake 

In this study, the weight of rats in each group increased with age. There was no significant difference in body weight between fructose and/or BPA exposed rats and control rats. Compared with control group, the food intake of 13% fructose, 20% fructose and combined exposure groups was significantly reduced, and the water intake of 13% fructose and combined exposure groups was significantly increased. Food and water intake of 1 µg/mL BPA group, and water intake of 20% fructose group were not different significantly from controls. The protein intake of 13% fructose, 20% fructose and combined exposure groups was significantly lower than that of control group. However, there was no difference in protein intake between 1 µg/mL BPA group and control group (Table 1).

### 3.2. Effects of Fructose and/or BPA Exposure on Energy Intake

Total energy intake of rats was computed based on food and water intake, as water containing fructose can provide energy. Compared with control group, 13% fructose, 20% fructose and combined exposure groups had significantly lower energy intake from food, while when added to the energy intake from water, 13% fructose and combined exposure groups had significantly higher total energy intake. However, compared with control group, the total energy intake of 20% fructose group and 1 µg/mL BPA group had no significant difference (Table 2).

### 3.3. Effects of Fructose and/or BPA Exposure on Visceral Fat and Liver Index

**Figure 1:**
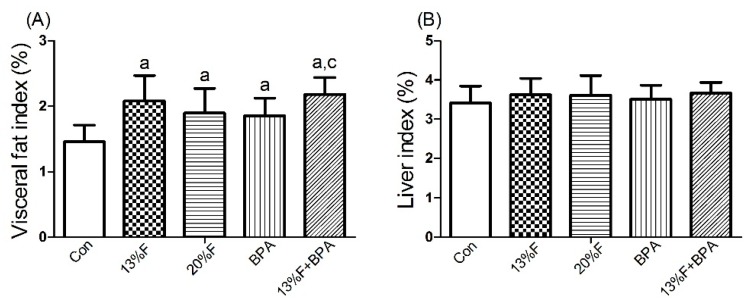
Effects of fructose and/or BPA exposure on index. (**A**) Visceral fat index; (**B**) Liver index of male rats after eight weeks of treatment with fructose and/or BPA. Values are expressed as means ± SD, *n* = 10. Differences between groups were considered as significant at *p* < 0.05 and were analyzed with one-way ANOVA. ^a^ different compared to Con; ^b^ different compared to 13% F; ^c^ different compared to BPA (*p* < 0.05). Con, control group; F, fructose group; BPA, 1 µg/mL BPA group.

The weight of visceral fat and liver were measured and the index was computed. The visceral fat coefficient in the experimental groups was higher than that in the control group. There was no significant difference in liver index between fructose and/or 1 µg/mL BPA exposure groups and controls (Figure 1).

### 3.4. Effects of Fructose and/or BPA Exposure on Serum Biochemistry

**Figure 2:**
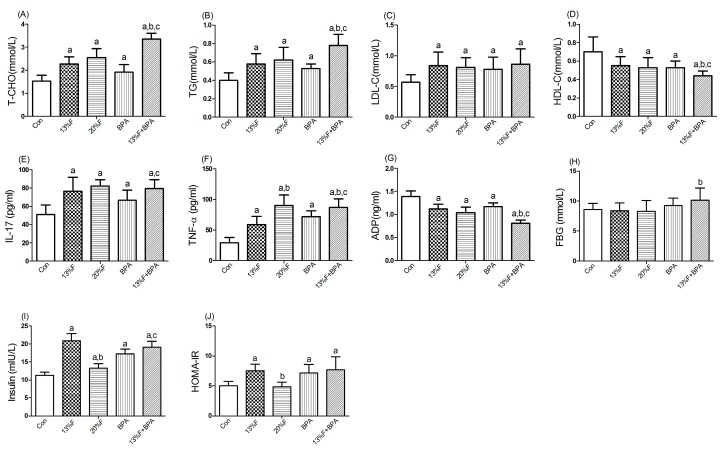
Effects of fructose and/or BPA exposure on lipid profile, inflammatory cytokines and ADP. Serum levels of (**A**) T-CHO; (**B**) TG; (**C**) LDL-C; (**D**) HDL-C; (**E**) IL-17; (**F**) TNF-α; (**G**) ADP; (**H**) FBG; (**I**) insulin; (**J**) HOMA-IR of male rats after eight weeks of treatment with fructose and BPA. Values are expressed as means ± SD, *n* = 10. Differences between groups were considered as significant at *p* < 0.05 and were analyzed with one-way ANOVA. ^a^ different compared to Con; ^b^ different compared to 13% F; ^c^ different compared to BPA (*p* < 0.05). Con, control group; F, fructose group; BPA, 1 µg/mL BPA group.

Compared with the control group, there were significantly higher levels of serum T-CHO, TG, LDL-C, IL-17 and TNF-α, and lower levels of serum HDL-C and ADP in all fructose and/or 1 µg/mL BPA exposure groups. More importantly, combined exposure to fructose and BPA had a synergistic effect on increasing T-CHO, TG and TNF-α, and decreasing HDL-C and ADP levels. There was no significant difference in FBG level in fructose and/or 1 µg/mL BPA exposure groups compared with controls. Serum levels of insulin in fructose and BPA alone or combined exposure groups were higher than those of control group. Except for 20% fructose group, the HOMA-IR of the rats treated with 13% fructose and BPA alone or in combination was statistically higher than that of control group (Figure 2). 

### 3.5. Liver Tissue Morphology

**Figure 3:**
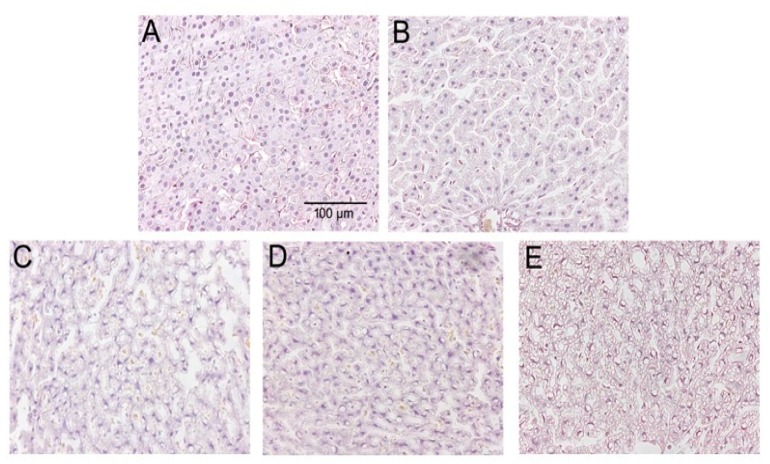
HE staining of liver tissues from five studied groups. (**A**) HE staining in Con group. (**B**) HE staining in 13% F group. (**C**) HE staining in 20% F group. (**D**) HE staining in BPA group. (**E**) HE staining in 13% F + BPA group. Original magnification 200×. Con, control group; F, fructose group; BPA, 1 µg/mL BPA group.

The morphology of liver tissue showed a normal hepatocyte structure with no degeneration and necrosis of cells after H&E staining in control group. However, the hepatocytes were condensed, and the cytoplasm was diffused, and more lipid droplets were clearly visualized in the liver tissue in fructose and/or BPA exposure groups. In addition, with the increase of fructose concentration, more and more lipid droplets were seen in the combined exposure group than the 13% fructose group and 1 µg/mL BPA group, indicating that the combined exposure group had more fat accumulation in the liver. Liver histomorphology showed that there were significant differences in the number of lipid droplets among the groups (Figure 3).

### 3.6. Effects of Fructose and/or BPA Exposure on the Expression of Genes Involved in Lipid Metabolism in Liver

**Figure 4:**
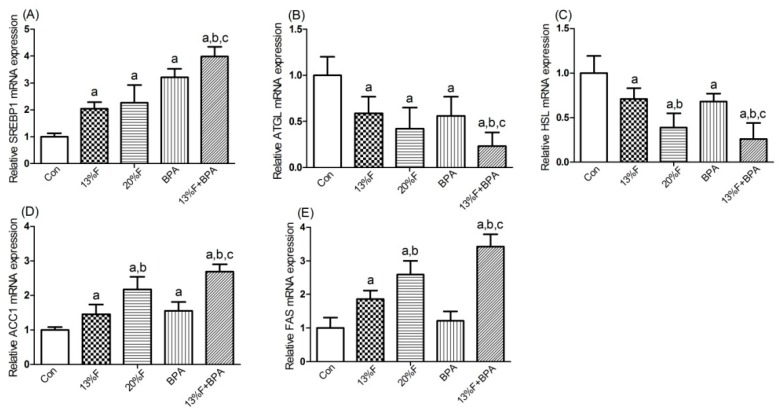
Effects of fructose and/or BPA exposure on the expression of genes involved in lipid metabolism in liver. Gene expression (qPCR) of (**A**) *SREBP1*; (**B**) *ATGL*; (**C**) *HSL*; (**D**) *ACC1*; (**E**) *FAS* in male rats after eight weeks of treatment with fructose or/and BPA. Values are expressed as means ± SD, *n* = 6. Differences between groups were considered as significant at *p* < 0.05 and were analyzed with one-way ANOVA. ^a^ different compared to Con; ^b^ different compared to 13% F; ^c^ different compared to BPA (*p* < 0.05). Con, control group; F, fructose group; BPA, 1 µg/mL BPA group.

As shown in Figure 4, *SREBP1* and *ACC1* mRNA levels were significantly elevated in fructose and/or BPA exposure groups compared with control group, and the levels of *SREBP1* and *ACC1* mRNA in combined exposure group were significantly higher than those in the single exposure group (Figure 4A,D). Compared with control group, the levels of *ATGL* and *HSL* mRNA in fructose and/or BPA exposure groups were significantly reduced (Figure 4B,C). There were significantly higher levels of *FAS* (Figure 4E) mRNA in all fructose exposure groups than in controls, but not in BPA exposure group.

### 3.7. Effects of Fructose and/or BPA Exposure on the Expression of Proteins Involved in Lipid Metabolism and Inflammatory Response in Liver

**Figure 5:**
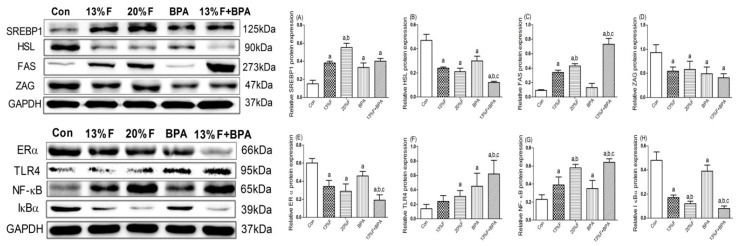
Hepatic protein levels in rats were analyzed by western blot, respectively. GAPDH protein was used as a control. (**A**) SREBP1 protein level. (**B**) HSL protein level. (**C**) FAS protein level. (**D**) ZAG protein level. (**E**) ERα protein level. (**F**) TLR4 protein level. (**G**) NF-κB protein level. (**H**) IκBα protein level. Values are expressed as means ± SD, *n* = 4. Differences between groups were considered as significant at *p* < 0.05 and were analyzed with one-way ANOVA. ^a^ different compared to Con; ^b^ different compared to 13% F; ^c^ different compared to BPA (*p* < 0.05). Con, control group; F, fructose group; BPA, 1 µg/mL BPA group.

The results showed that the levels of SREBP1 protein significantly increased, HSL, ERα and ZAG protein significantly decreased in fructose and/or BPA exposure groups, and FAS protein significantly increased in 13% fructose, 20% fructose and combined exposure groups compared with control group. There was no significant difference in FAS protein expression between BPA and control group. The levels of TLR4 and NF-κB protein in the liver of rats exposed to fructose and/or BPA were significantly higher, while the levels of IκBα protein were significantly lower than that of the control group. In addition, there were synergetic effects on increased FAS, TLR4 and NF-κB protein, and decreased HSL, ERα and IκBα protein in combined 13% fructose and BPA exposure compared with single 13% fructose or BPA exposure (Figure 5). 

## 4. Discussion

Considering that fructose and BPA often coexist in foods or beverages, and children and adolescents are the main consumers of these sugary products, this study aimed to explore whether fructose and BPA have synergistic effects on abnormal lipid metabolism and its possible mechanism in developmental rats. In this study, 13% and 20% fructose were used as low and high doses respectively, and 1 µg/mL BPA was used as low dose according to the reference [21,22]. Our results showed that abnormal lipid metabolism and inflammatory response were more serious in combined fructose and BPA exposed rats. The current study, to the best of our knowledge, is the first report revealing synergistic adverse effects of combined exposure to fructose and BPA on lipid metabolism.

In this study, the average water intake was 30.70 and 64.70 mL/rat/day, while the average weight was 263.00 and 270.35 g in BPA group and combined exposure group, respectively. We estimated that the average BPA exposure levels of the two groups during the experiment were approximately 116.73 and 239.32 µg/kg BW/day, respectively, which corresponded to human exposure levels 18.93 and 38.81 µg/kg BW/day, respectively, according to the dose conversion method from animal to human reported described by Reagan-Shaw et al [23]. In addition, bottle spillage may result in the actual BPA exposure level to be lower than we calculated. Therefore, the dose of BPA used in this study was lower than the lowest observable adverse effect level (LOAEL) set by the United States Environmental Protection Agency (EPA) (50 µg/kg BW/day). The water intake of rats in 13% fructose and combined exposure groups increased significantly, and the food intake decreased significantly, suggesting that 13% fructose sweetness is more suitable for rats, which can stimulate rats to increase water intake and obtain more energy. Janevski et al. also reported similar results [24]. Although there was no significant difference in water intake between 20% fructose group and control group, total energy intake was also significantly higher in 20% fructose group. These results implied that the intake of SSBs leads to a reduction in food intake and an accompanied reduction in protein intake, suggesting that excessive SSBs intake may have adverse effects on growth and development. 

There was no significant difference in body weight among fructose and/or BPA exposed rats and control rats. At present, the effect of fructose or BPA exposure alone on body weight is inconsistent, showing no change in body weight, increase or decrease [11,25,26,27]. The contradictory results may be related to animal species, age and sex, dosage, route and time of fructose or BPA exposure and other factors. However, we found that the visceral fat coefficient in fructose and/or BPA groups was significantly higher than that in the control group. Studies have found that many thin people have more visceral adipose tissue than overweight or obese people, a phenomenon known as “thin on the outside, fat on the inside” (TOFI) [28,29]. Metabolic obese normal weight (MONW) refers to normal body weight but presents with a range of obesity-related diseases, so measuring body weight alone does not effectively identify the risk of metabolic syndrome due to increased visceral adipose tissue [30]. Other studies have also found that exposure to fructose or BPA can lead to visceral adiposity, metabolic disorders, cardiovascular disease without body weight change [29,31,32,33]. 

In recent year, there have been many studies on the relationship between fructose exposure and abnormal lipid metabolism, but few studies on the effect of BPA on blood lipids and key enzymes regulating lipid metabolism. A recent meta-analysis found that consuming fructose, rather than glucose-containing beverages led to visceral adiposity, increased serum TG levels, elevated systolic blood pressure, hyperglycaemia and hyperinsulinemia [34]. The levels of serum TG, T-CHO and LDL-C increased, and HDL-C decreased in 10% fructose-fed rats [35]. BPA administration resulted in higher levels of serum TG, T-CHO and LDL-C [36]. ADP is an important adipocytokine, which plays a vital role in glucose and lipid metabolism, inflammation and oxidative stress, and its reduction has been demonstrated to directly increase the synthesis of lipids, free fatty acids and inflammatory cytokines [37]. A recent research reported that maternal and/or paternal high-fructose diet had adverse effects on liver metabolism with decreased ADP levels in their offspring adult male mice [38]. Our previous study showed that obesity and dyslipidemia induced by perinatal exposure to BPA were associated with down-regulation of ADP [39]. In this study, combined exposure to fructose and BPA had a synergistic effect on increasing T-CHO and TG, and decreasing HDL-C and ADP levels.

IR is characterized by a decrease in the effect of insulin despite an increase in insulin concentration, and is associated with type 2 diabetes, hypertension, atherosclerosis, NAFLD and so on [40,41]. In our study, serum insulin levels and HOMA-IR were elevated in rats exposed to fructose and/or BPA, although FBG levels did not change significantly compared with controls. High levels of insulin promote lipogenesis, because insulin can induce the synthesis of 3-hydroxy-3-methylglutarylcoenzyme a (HMG-CoA) reductase in the liver, a rate-limiting enzyme in cholesterol synthesis, which is consistent with increased gene and protein expression of lipogenesis in the liver of fructose and/or BPA exposed rats. In addition, the levels of *SREBP1*, *ACC1* and *FAS* genes were the highest, while *ATGL* and *HSL* genes were the lowest in the liver of rats exposed to both fructose and BPA. In our study, the level of *SREBP1* gene was the highest, but its protein expression was not the highest in the liver of combined BPA and fructose exposed rats. The reason may be that the process of gene translation to protein is complex, and the expression of protein may be affected by histone modification (epigenetic modification), which needs further study. Our results were in accordance with the findings by Ronn et al., which indicated that BPA exposure increased liver fat in juvenile fructose-fed Fischer 344 rats [42]. Studies showed that 10% fructose-fed rats for 5 weeks increased *FAS* mRNA expression and correspondingly decreased *HSL* mRNA expression [43]. Low doses of BPA (5, 50, 500 µg/kg/day), rather than a higher dose (5000 µg/kg/day), induced genes expression associated with lipid synthesis, including *ACC1* and *FAS*, and triggered TG accumulation in the liver of adult mice [44]. Our results indicate that combined exposure to fructose and BPA has a synergistic effect on increasing lipogenic enzymes and decreasing lipolytic enzymes, which is consistent with the more lipid droplets in rat liver tissue.

ZAG is a novel type of adipokine involved in adipose tissue mobilization, which plays an important role in regulating lipid metabolism, energy balance and maintaining body mass. Decreased levels of ZAG are related to diminished lipolysis [45]. Structurally, BPA can mimic estrogen binding to ER, resulting in additional estrogen effects and possibly estrogen-related diseases [46]. ERα signaling pathway has many physiological functions, one of which is to regulate lipid metabolism. For example, ERα can improve hepatic metabolism and inhibit lipid deposition in the liver during the regulation of hepatic lipid metabolism [47]. Moreover, increased levels of *FAS* and *ACC1* mRNA in male ERα knockout mice were observed to lead to increased liver lipid deposition and TG levels [48]. In our study, ZAG and ERα protein levels were the lowest in the liver of combined fructose and BPA exposed rats, further confirming the synergistic effect of fructose and BPA on decreasing lipolysis and increasing lipogenesis. These findings showed that the combined exposure to fructose and BPA could exacerbate abnormal lipid metabolism in developmental rats. 

At present, the mechanism of inflammatory response promoting abnormal lipid metabolism is still unclear. In general, IR can promote the decomposition of fat and the production of excessive free fatty acid (FFA), which can active the production of inflammatory cytokines, such as IL-6 and TNF-α, through TLR4/NF-κB signaling pathway, leading to enhanced inflammatory response. While, excessive inflammatory cytokines can activate NF-κB, and cause IR. IR can induce lipid metabolism disorders. Hypercholesterolemia also can lead to an enhanced immune response to modified lipids and aortic inflammation, increased Th17 cells in the spleen, and a positive correlation between progression of atherosclerosis and Th17 cells [49]. TLR4 is one of the pattern recognition receptors, which plays an important role in initiating inflammatory response and activating the down-streaming signaling NF-κB [50]. Activation of NF-κB is accompanied by the degradation of IκBα and further induces the expression of inflammatory cytokines IL-6 and TNF-α [51,52]. Studies have found that magnesium isoglycyrrhizinate inhibits the activation of hepatic NF-κB under fructose exposure, accompanied by the decrease in the levels of inflammatory cytokines, thereby regulating lipid metabolism and attenuating hepatic lipid accumulation [17]. Mahmoudi et al. found that the extract of olive leaves has the effect of reducing blood lipid and ameliorating liver damage by regulating the activity of NF-κB signaling pathway [9]. Our results showed that there were the highest levels of TLR4 and NF-κB protein in the liver of combined fructose and BPA exposed rats, which indicated that there were also synergistic effects of combined fructose and BPA exposure on activating inflammatory response. Therefore, combined exposure to fructose and BPA can exacerbate adverse health effects, compared with fructose or BPA exposure alone. Our results provide a basis for establishing environmental exposure threshold affecting human health, and the interaction between environmental factors should be considered.

## 5. Conclusions

In conclusion, our study showed that there were synergistic effects of combined exposure to fructose and BPA on abnormal lipid metabolism, which were associated with increasing lipogenic enzymes, decreasing lipolytic enzymes, and activating inflammatory response in liver of developmental male rats. Therefore, combined exposure to fructose and BPA could exacerbate abnormal lipid metabolism and lead to more fat accumulation in liver, compared with fructose or BPA exposure alone.

## Figures and Tables

**Table 1:** The effect of fructose and/or BPA exposure on body weight, and food, water and protein intake in male rats.

Group	Body Weight (g)		Food Intake (g/rat/day)		Water Intake (mL/rat/day)		Protein Intake (g/rat/day)	
	Month 1	Month 2	Month 1	Month 2	Month 1	Month 2	Month 1	Month 2
Con	224.20 ± 50.59	300.60 ± 54.33	17.75 ± 1.90	23.53 ± 1.16	23.60 ± 1.78	31.90 ± 2.23	4.23 ± 0.65	5.60 ± 0.54
13% F	231.30 ± 32.71	305.20 ± 43.06	12.83 ± 1.80^a^	15.02 ± 0.86^a^	54.10 ± 7.00^a^	78.60 ± 4.45^a^	3.07 ± 0.62^a^	3.47 ± 0.47^a^
20% F	225.50 ± 27.41	302.40 ± 44.05	12.66 ± 0.54^a^	15.93 ± 1.61^a^	26.60 ± 2.55^b^	34.30 ± 6.15^b^	3.01 ± 0.48^a^	3.76 ± 0.61^a^
BPA	224.60 ± 61.93	301.40 ± 76.63	17.82 ± 1.67	23.91 ± 2.32	26.30 ± 4.37	35.10 ± 3.96	4.23 ± 0.60	5.35 ± 0.66
13% F + BPA	233.00 ± 37.06	307.70 ± 58.54	13.78 ± 0.67^ac^	15.64 ± 1.19^ac^	51.00 ± 5.73^ac^	78.40 ± 3.34^ac^	3.25 ± 0.49^ac^	3.67 ± 0.54^ac^

Values are expressed as means ± SD, *n* = 10. Differences between groups were considered as significant at *p* < 0.05 and were analyzed with one-way ANOVA. ^a^ different compared to Con; ^b^ different compared to 13% F; ^c^ different compared to BPA (*p* < 0.05). Con, control group; F, fructose group; BPA, 1 µg/mL BPA group.

**Table 2:** The effect of fructose and/or BPA exposure on energy intake in male rats (kcal/rat/day).

Group	Month 1			Month 2		
	Food	Water	Total	Food	Water	Total
Con	57.48 ± 6.03	−	57.48 ± 6.03	76.33 ± 3.48	−	76.33 ± 3.48
13% F	41.55 ± 5.69^a^	28.17 ± 3.69	69.72 ± 4.34^a^	47.36 ± 1.19^a^	40.73 ± 2.55	88.09 ± 1.76^a^
20% F	41.05 ± 1.21^a^	21.47 ± 2.41^b^	62.42 ± 1.87^b^	51.57 ± 5.03^a^	27.60 ± 5.21^b^	79.14 ± 2.62^b^
BPA	57.79 ± 5.24	−	57.79 ± 5.24	77.49 ± 7.43	−	77.49 ± 7.43
13% F + BPA	44.60 ± 1.73^ac^	26.52 ± 3.13	71.12 ± 1.71^ac^	50.69 ± 3.65^ac^	40.78 ± 2.01	91.50 ± 2.97^ac^

Values are expressed as means ± SD, *n* = 10. Differences between groups were considered as significant at *p* < 0.05 and were analyzed with one-way ANOVA. ^a^ different compared to Con; ^b^ different compared to 13% F; ^c^ different compared to BPA (*p* < 0.05). Con, control group; F, fructose group; BPA, 1 µg/mL BPA group.
